# Phenformin and ataxia‐telangiectasia mutated inhibitors synergistically co‐suppress liver cancer cell growth by damaging mitochondria

**DOI:** 10.1002/2211-5463.13152

**Published:** 2021-04-03

**Authors:** Tianyu Wu, Sichun Zhou, Mei Qin, Jing Tang, Xinjian Yan, Lingli Huang, Meiyuan Huang, Jun Deng, Di Xiao, Xin Hu, Jingtao Wu, Xiaoping Yang, Gaofeng Li

**Affiliations:** ^1^ Department of Oncology Zhuzhou Hospital Affiliated to Xiangya School of Medicine Central South University Zhuzhou China; ^2^ Key Laboratory of Protein Chemistry and Developmental Biology of Fish of Ministry of Education Key Laboratory of Study and Discovery of Small Targeted Molecules of Hunan Province Department of Pharmacy School of Medicine Hunan Normal University Changsha China; ^3^ Department of Gynecologists Zhuzhou Hospital Affiliated to Xiangya School of Medicine Central South University Zhuzhou China; ^4^ Department of Pathology Zhuzhou Hospital Affiliated to Xiangya School of Medicine Central South University Zhuzhou China

**Keywords:** KU‐55933, liver cancer, mitochondria, p‐AMPK, phenformin

## Abstract

Inhibitors of ataxia**–**telangiectasia mutated (ATM), such as KU‐55933 (Ku), represent a promising class of novel anticancer drugs. In addition, the biguanide derivative phenformin exhibits antitumor activity superior to that of the AMPK activator metformin. Herein, we assessed the potential combinatorial therapeutic efficacy of phenformin and Ku when used to inhibit the growth of liver cancer cells, and we assessed the mechanisms underlying such efficacy. The Hep‐G2 and SMMC‐7721 liver cancer cell lines were treated with phenformin and Ku either alone or in combination, after which the impact of these drugs on cellular proliferation was assessed via 3‐(4,5‐dimethylthiazol) 2, 5‐diphenyltetrazolium and colony formation assays, whereas Transwell assays were used to gauge cell migratory activity. The potential synergy between these two drugs was assessed using the compusyn software, while flow cytometry was employed to evaluate cellular apoptosis. In addition, western blotting was utilized to measure p‐ATM, p‐AMPK, p‐mTOR, and p‐p70s6k expression, while mitochondrial functionality was monitored via morphological analyses, JC‐1 staining, and measurements of ATP levels. Phenformin and Ku synergistically impacted the proliferation, migration, and apoptotic death of liver cancer cells. Together, these compounds were able to enhance AMPK phosphorylation while inhibiting the phosphorylation of mTOR and p70s6k. These data also revealed that phenformin and Ku induced mitochondrial dysfunction as evidenced by impaired ATP synthesis, mitochondrial membrane potential, and abnormal mitochondrial morphology. These findings suggest that combination treatment with phenformin and Ku may be an effective approach to treating liver cancer via damaging mitochondria within these tumor cells.

## Graphical abstract

Phenformin and ku‐55933 have synergistic effects on inhibition of proliferation, migration, and apoptosis of liver cancer cell lines. These two compounds stimulate p‐AMPK protein and inhibit the expression of p‐mTOR and p‐p70s6k. Furthermore, phenformin and ku‐55933 induced mitochondrial dysfunction as evidenced by decreased ATP synthesis, mitochondrial membrane potential, and disrupted mitochondrial morphology.

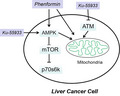

**Abbreviations**
AMP: adenosine monophosphate
AMPK: adenosine 5’‐monophosphate (AMP)‐activated protein kinase
ATM: ataxia–telangiectasia mutated
MMP: mitochondrial membrane potential
mTOR: mammalian target of rapamycin
MTT: 3‐(4,5‐dimethylthiazol) 2, 5‐diphenyltetrazolium

Liver cancer is the fifth most common cancer subtype. While it can be treated via surgery or radiotherapy, outcomes in patients are often unsatisfactory owing to the high rates of invasion and recurrence [1]. Drug‐based treatment strategies are thus an attractive alternative approach to liver cancer management, and several promising therapeutic candidates with fewer side effects than those associated with conventional chemotherapeutic regimens have emerged in recent years. However, as liver tumors rapidly acquire drug resistance, there is a clear need for the development of novel compounds that can be used to specifically treat this form of cancer. In prior preclinical analyses, phenformin was shown to be very safe [2], while also functioning in a manner similar to metformin, functioning by impairing cancer cell proliferation, and promoting cell cycle arrest and apoptotic death [3]. Single‐agent phenformin treatment, however, is not an appropriate approach owing to its potential to induce severe lactic acidosis [4]. The application of two or more drugs can often achieve better outcomes than single‐agent therapy owing to multimodal antitumor activity that can reduce the rate of drug resistance while preventing tumor progression or metastasis [5]. Indeed, multiple studies have found that phenformin can be used in combination with a range of other anticancer agents as a means of treating different tumor types [6, 7, 8, 9]. Combination therapy enables clinicians to deliver significantly lower phenformin doses that are not associated with potentially fatal lactic acidosis. Ataxia–telangiectasia mutated (ATM) is a regulator of many developmental processes within tumor cells including migration, metabolic regulation, cell cycle checkpoint regulation, and DNA double‐strand break repair [10]. KU‐55933 (Ku) is an ATM inhibitor that has been used to treat a variety of cancers [11], functioning by inducing mitochondrial superoxide production and thereby synergistically driving apoptotic cell death [12]. Prior work led us to hypothesize that the ATM inhibitor Ku and the AMPK activator phenformin may offer synergistic therapeutic advantages when used to treat liver cancer. Consistent with this hypothesis, we herein demonstrate that phenformin and Ku synergistically function to suppress liver cancer cell growth via enhancing AMPK activity and damaging mitochondria within these tumor cells.

## Materials and methods

### Reagents

Phenformin (Aladdin Chemistry, Shanghai, China) was reconstituted at 100 mm in PBS. KU‐55933 (Selleck‐Biotool, S1092, Shanghai, China) was reconstituted at 30 mm in DMSO. Both compounds were diluted to appropriate working concentrations using cell culture medium. Rabbit anti‐human antibodies specific for the following proteins were obtained from Cell Signaling (Massachusetts, USA): phospho‐p70s6k kinase (Tr389), total AMPKα, phospho‐AMPKα (Tr172), phospho‐mTOR (Ser2448), and β‐actin. Antibodies against total ATM and phospho‐ATM (Ser1987) were obtained from Abcam (Cambridgeshire, UK). All antibodies were diluted to a working concentration of 1 : 1000.

### Cell culture

Human Hep‐G2 and SMMC‐7721 liver cancer cells were obtained from the School of Medicine Hunan Normal University (Changsha, Hunan, China) and were cultured in Dulbecco's modified Eagle's medium (HyClone, Logan, UT, USA) containing 10% FBS (HyClone, Logan, UT, USA) and penicillin/streptomycin (HyClone, Logan, UT, USA).

### MTT assay

An 3‐(4,5‐dimethylthiazol) 2, 5‐diphenyltetrazolium (MTT) assay was used to evaluate the viability of liver cancer cells. Briefly, cells were plated at 8 × 10^3^/well in 96‐well plates for 24 h, after which they were treated for 72 h with a range of Ku and phenformin doses. First, the IC50 values of phenformin and Ku in the SMCC‐7721 or Hep‐G2 cell lines were determined via MTT assay. Appropriate low concentrations were then selected for combination treatment to detect whether these agents exhibited synergistic efficacy. MTT tetrazolium salt (Sigma, MO, USA) was then added to each well and plates were incubated for 5 h, after which 150 μL of DMSO (Sigma) was added to each well, and absorbance at 490 nm was assessed via microplate reader (BioTek, Synergy HTX, VT, USA).

### Colony formation assay

Liver cancer cell proliferation was monitored via colony formation assay. Briefly, cells were plated at 7 × 10^3^/well in 24‐well plates for 24 h, after which they were treated with a range of Ku and phenformin doses. SMMC‐7721 cells were divided into four treatment groups: PBS, Ku (5 μm), phenformin (80 μm), or combination phenformin (80 μm) and Ku (5 μm). Similarly, Hep‐G2 cells were divided into four treatment groups: PBS, Ku (5 μm), phenformin (40 μm), or combination of phenformin (40 μm) and Ku (5 μm). After 7 days, absorbance at 550 nm was assessed via microplate reader, and images were collected via microscopy.

### Transwell migration assay

A Transwell assay was employed to assess cellular migration. Briefly, 6 × 10^4^ liver cancer cells were added to the upper chamber of a Transwell system, with media containing 10% FBS being added to the lower chamber. SMMC‐7721 cells were divided into four treatment groups: PBS, Ku (10 μm), phenformin (80 μm), or phenformin (80 μm) + Ku (10 μm). Similarly, Hep‐G2 cells were divided into four treatment groups: PBS, Ku (10 μm), phenformin (60 μm), or phenformin (60 μm) + Ku (10 μm). Following a 24‐h incubation, cells on the upper surface were removed, and those that had migrated to the lower surfaces were fixed using 10% formaldehyde followed by staining using 0.1% crystal violet. Cells in three random fields of view per well were imaged and counted.

### Assessment of cellular apoptosis

An Annexin V‐FITC/PI dual‐staining assay (Beyotime, Shanghai, China) was used to gauge apoptotic cell death. Briefly, liver cancer cells were plated in 6‐well plates at 6 × 10^5^/well and were treated for 12 or 24 h with a range of Ku and phenformin doses. SMMC‐7721 cells were divided into four treatment groups: PBS, Ku (10 μm), phenformin (400 μm), or phenformin (400 μm) + Ku (10 μm). Similarly, Hep‐G2 cells were divided into four treatment groups: PBS, Ku (10 μm), phenformin (200 μm), or phenformin (200 μm) + Ku (10 μm). Cells were then harvested and stained for 20 min in a 300 μL volume containing 5 µL each of Annexin V‐APC and PI, protected from light. Stained cells were then assessed with a BD FACSCanto flow cytometer.

### ATP consumption assay

An ATP Detection Kit (Nanjing Jiancheng Bioengineering Institute, China) was used based on provided directions to assess ATP levels in liver cancer cells. Briefly, cells were added to 6‐well plates (7 × 10^5^/well) and were treated for 24 h with Ku (10 μm), phenformin (200 μm), or a combination of the two. After following kit protocols, absorbance at 490 nm was measured, and ATP content per unit was calculated with an appropriate formula: ATP concentration (μmol/g prot) = (Sample OD‐Control OD) /(Standard OD‐Blank OD)*Standard Sample Concentration (1 × 10^3^ μm)* Sample dilution ratio/Sample protein concentration.

### Mitochondrial membrane potential analysis

A JC‐1 Staining Kit (Solarbio Life Sciences, Beijing, China) was used to measure mitochondrial membrane potential (MMP). Briefly, 2 × 10^5^ cells were added to 12‐well plates and treated for 24 h with Ku (10 μm), phenformin (200 μm), or a combination of the two. Cells were then treated with a JC‐1 working solution, followed by two washes with JC‐1 buffer, after which cells were imaged via fluorescence microscopy.

### Assessment of mitochondrial morphology

Following a 24 h treatment period with Ku (10 μm), phenformin (200 μm), or a combination of the two, 6 × 10^6^ cells were collected, washed two times using PBS, digested with trypsin, and fixed with cold 3% glutaraldehyde in PBS at 4 °C for 24 h. Samples were next postfixed using 1% osmium tetroxide, dehydrated using an ethanol gradient (30–90%), and embedded in 812 Epoxy Resin. Ultrathin sections (80 nm) were then stained using 2% uranic acetate and lead citrate, followed by imaging with an H7650 transmission electron microscope (TEM; Hitachi, Tokyo, Japan).

### Western blotting

Proteins were first separated and transferred to appropriate membranes, which were then stained for 12–15 h using the primary antibodies described above. Blots were then washed using TBS, probed with secondary antibodies for 2 h, and washed four more times prior to imaging with a ChemiDoc system (Bio‐Rad, CA, USA). Films were scanned using Tanon‐4500, and densitometric analyses were conducted with the imagej software (Rawak Software Inc, Stuttgart, Germany).

### Statistical analyses

Data were compared via two‐tailed *t*‐tests or two‐way ANOVAs, as appropriate. *P* < 0.05 served as the significance threshold for this study. *P < 0.05; **P < 0.01; ***P < 0.001.

## Results

### Phenformin and Ku synergistically suppress liver cancer cell viability

**Fig. 1:**
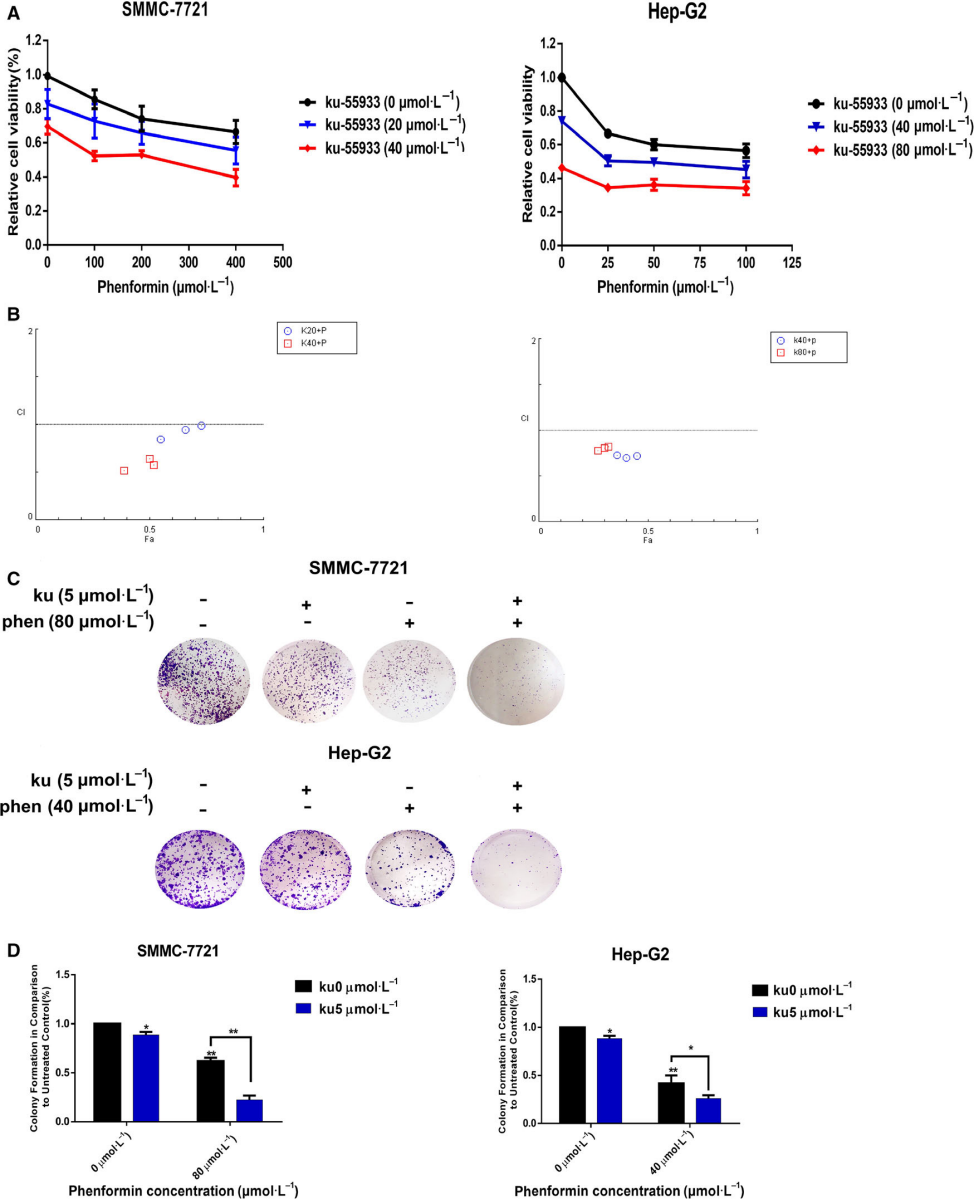
The impact of phenformin and Ku on SMMC‐7721 and Hep‐G2 cell proliferation and colony formation. (A) Phenformin together with Ku inhibited SMMC‐7721 and Hep‐G2 proliferation synergistically. SMMC‐7721 and Hep‐G2 cell viability was assessed at 72 h post‐treatment with phenformin alone or in combination with Ku. (B) The combination index (CI) metric corresponding to interaction between the two drugs was calculated. CI = 1 indicates an additive interaction, CI > 1 indicates antagonism, and CI < 1 is indicative of synergism. CI values for nearly all combinations were less than 1, consistent with moderately strong synergism. (C) Evaluation of the ability of phenformin and Ku to suppress colony formation. Cells were treated for 7 days with phenformin and Ku either alone or in combination. (D) Quantification of the colony formation experiment is shown in (C). Wells were analyzed at 550 nm. Data are means ± SD of three independent experiments. *P < 0.05, ** *P* < 0.01 vs. control (two‐tailed *t*‐test).

We began by treating Hep‐G2 and SMMC‐7721 cells with a range of phenformin and/or Ku doses. Interestingly, we found that the sensitivity of these two cell lines to phenformin differed markedly, with distinct IC_50_ values and corresponding dose curves (Fig. S1). Hep‐G2 cells were more sensitive to phenformin than were SMMC‐7721 cells (Fig. 1A), and as such, different concentrations were used in subsequent assays for these two cell lines. However, the Ku IC_50_ was similar in both cell lines, and the same concentration of this inhibitor was therefore used in subsequent assays. Combination treatment with both of these drugs was better able to inhibit liver cancer cell growth than was single‐agent treatment with either of these drugs (Fig. 1A). Using the compusyn software, the drug combination index for these two agents was determined (Fig. 1B), revealing that phenformin and Ku functioned synergistically as evidenced by a CI < 1.

### Phenformin and Ku suppress liver cancer cell colony formation

The combinatorial antitumor activity of phenformin and Ku was also evaluated via colony formation assay. Dose curves (Fig. 1A) were used to select optimal doses of both of these agents in an effort to maximize antiproliferative activity (Fig. 1C,D). Combination treatment with phenformin and Ku markedly suppressed liver cancer cell colony formation in this assay system. We observed that Ku (5 μm), phenformin (80 μm), or both exhibited increasingly robust anticlonogenic effects, inhibiting colony formation by 11.4% ± 3.1%, 38.7% ± 3.5%, and 78.3% ± 4.5% in SMMC‐7721 cells, respectively, while Ku (5 μm), phenformin (40 μm), or both inhibited colony formation by 12.8% ± 2.9%, 56.8% ± 8.4%, and 75.9 ± 3.4%, respectively, in Hep‐G2 cells.

### Phenformin and Ku inhibit the migration of liver cancer cells

**Fig. 2:**
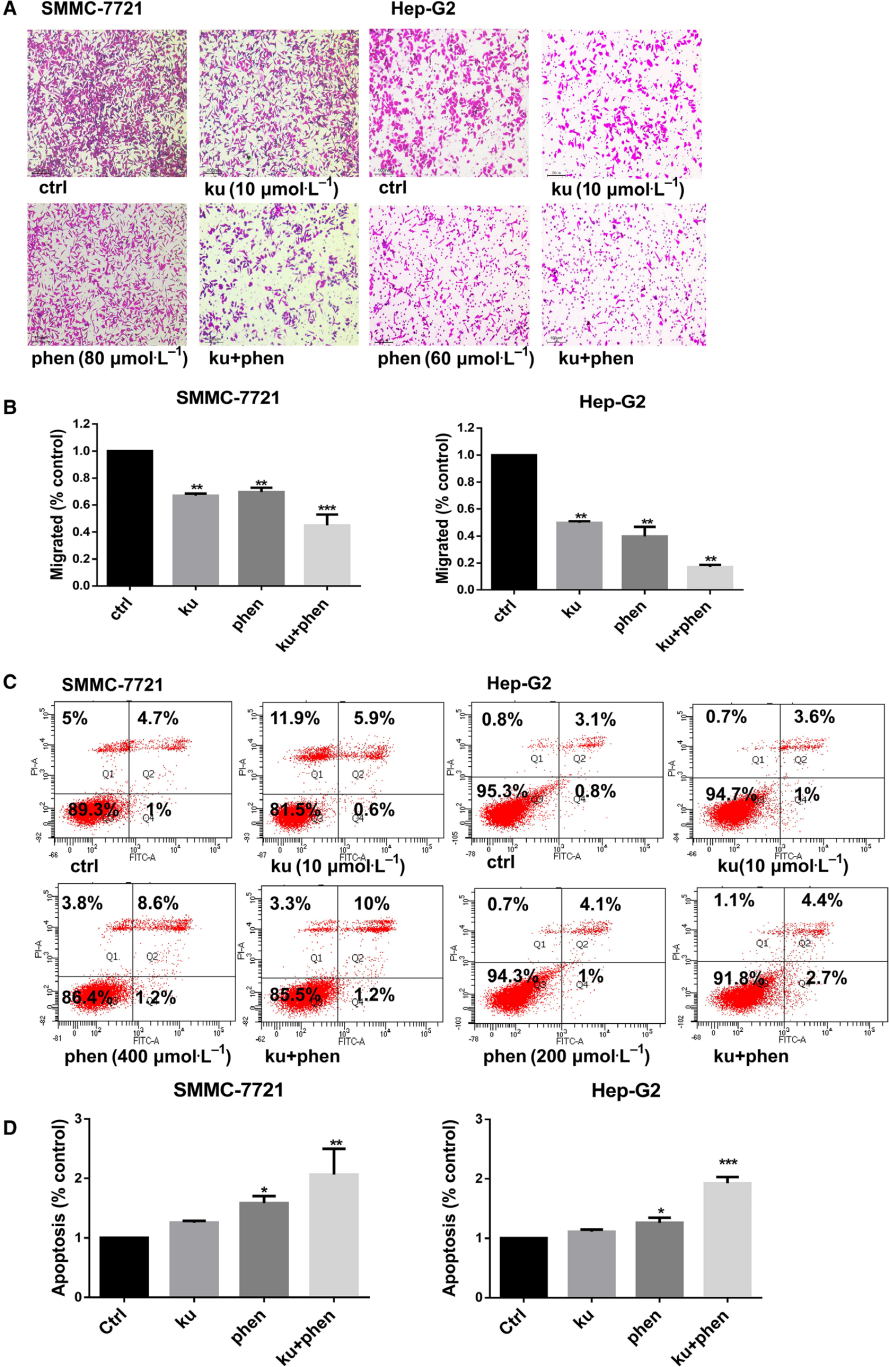
The impact of phenformin and Ku on migration and apoptosis in SMMC‐7721 and Hep‐G2 cells. (A) A combination of phenformin and Ku inhibited SMMC‐7721 and Hep‐G2 cell migration in a Transwell assay. SMMC‐7721 cells were treated with 80 μm phenformin alone, 10 μm Ku alone, or both for 24 h. Hep‐G2 cells were treated with 60 μm phenformin alone, 10 μm Ku alone, or both for 24 h. Scale bar, 100 μm. (B) Quantification of the results is shown in (A). (C) Representative flow cytometry scatter plots showing propidium iodide (*y*‐axis) and Annexin V‐FITC (*x*‐axis)‐stained cells. SMMC‐7721 cells were treated with 400 μm phenformin alone, 10 μm Ku alone, or both for 24 h. Hep‐G2 cells were treated with 200 μm phenformin alone, 10 μm Ku alone, or both for 24 h. (D) Quantification of flow cytometry results. Data are means ± SD, *n* = 3. *P < 0.05, **P < 0.01, ***P < 0.001 (one‐way ANOVA).

We next utilized Transwell assays to evaluate the impact of phenformin and Ku on SMMC‐7721 and Hep‐G2 cell migration. This approach revealed observed Ku (10 μm), phenformin (80 μm), or both suppressed migration by 30.5% ± 3.2%, 33.1% ± 1.5%, and 55.2% ± 8.1%, respectively, in SMMC‐7721, while Ku (10 μm), phenformin (60 μm), or both inhibited Hep‐G2 cell migration by 50.2% ± 1.2%, 60.2% ± 7.1%, and 83.2% ± 15.7%, respectively, thus confirming that combination treatment with both of these compounds inhibited migration more effectively than single‐agent treatment with either compound individually (Fig. 2A,B).

### Phenformin and Ku synergistically promote apoptotic cell death

Next, flow cytometry was used to gauge the impact of phenformin and Ku on liver cancer cell apoptosis. Relative to untreated cells, phenformin alone was able to enhance Hep‐G2 and SMMC‐7721 cell apoptosis, whereas Ku had no impact on the apoptotic death of these cells (Figs 2C,D and S2). Interestingly, combination treatment with phenformin and Ku induced significantly more apoptotic cell death than did phenformin alone, suggesting that Ku synergistically sensitizes liver cancer cells to phenformin‐induced death. Given that distinct cellular responses to phenformin were observed in proliferation, colony formation, Transwell, and apoptosis assays, we selected our doses based upon individual assay conditions. Higher doses were used in the apoptosis assay than in western blotting and other assays, in line with prior studies [13, 14]. We observed that treatment for 24 h with phenformin (400 μm) alone or in combination with Ku (10 μm) induced apoptosis rates of 1.58 ± 0.12 and 2.06 ± 0.43, respectively, in SMMC‐7721 cells, while phenformin (200 μm) alone or in combination with Ku (10 μm) induced apoptosis rates of 1.26 ± 0.09 and 1.92 ± 0.11, respectively, in Hep‐G2 cells (Fig. 2C,D). We also observed that 12 h treatment with phenformin (400 μm) alone or in combination with Ku (10 μm) induced apoptosis rates of 1.49 ± 0.11 and 2.48 ± 0.35, respectively, in SMMC‐7721 cells, while phenformin (200 μm) alone or in combination with Ku (10 μm) induced apoptosis rates of 1.70 ± 0.17 and 2.79 ± 0.22, respectively, in Hep‐G2 cells (Fig. S2).

### Ku activates AMPK *in vitro*

**Fig. 3:**
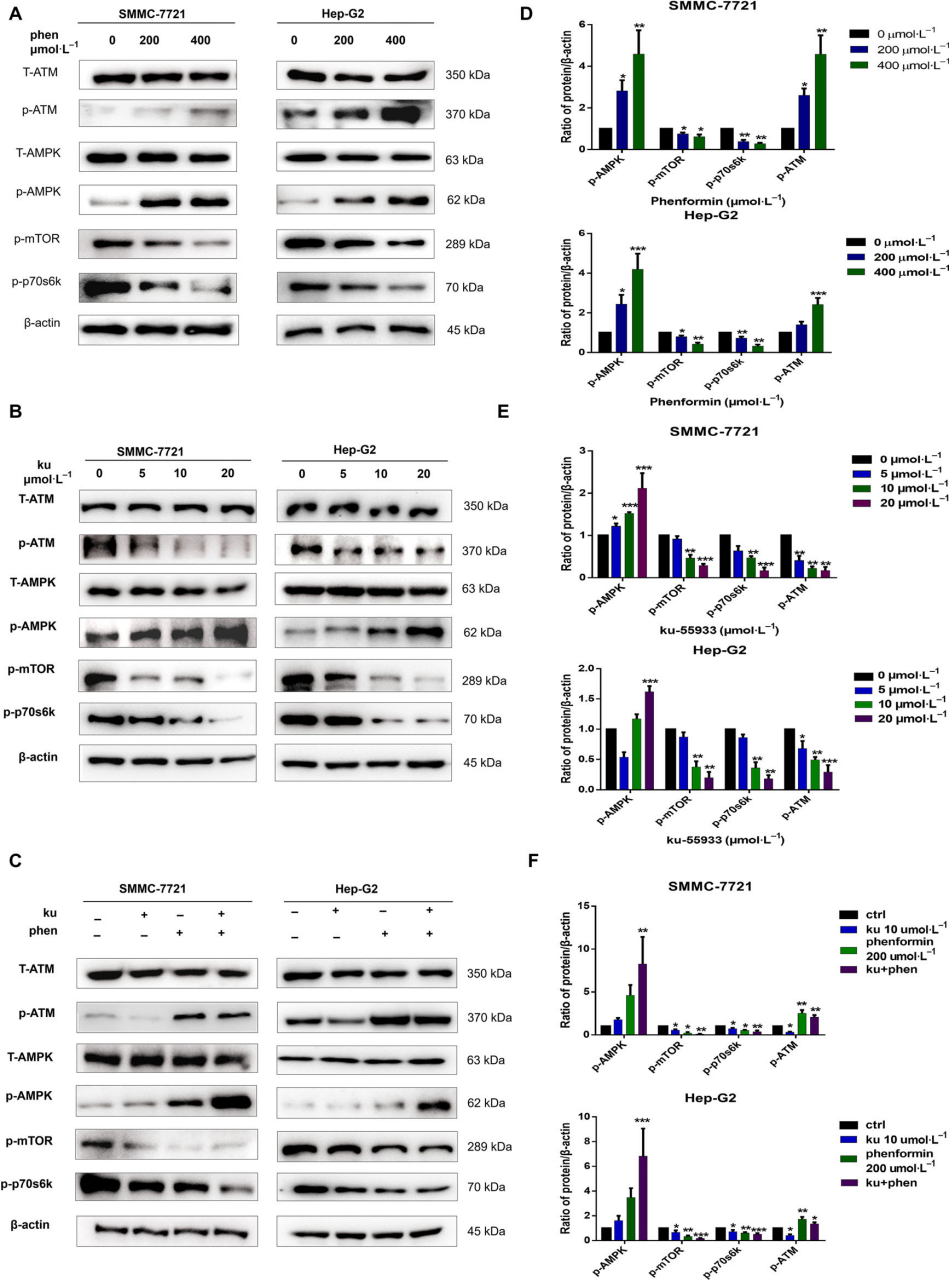
The impact of phenformin and Ku alone or in combination on AMPK signaling pathways. Western blotting was used to examine total (T) and phosphorylated (p) forms of various signaling proteins in SMMC‐7721 and Hep‐G2 cells treated with phenformin (0, 200, 400 μm), Ku (0, 5, 10, 20 μm), or both (200 μm phenformin + 10 μm Ku) for 24 h. Control cells received no drugs. (A–C) Western blots of p‐AMPK, t‐AMPK, p‐mTOR, p‐p70S6K, p‐ATM, and t‐ATM. β‐actin was included as a loading control. (D–F) Relative levels of various proteins. Data are means ± SD, *n* = 3. *P < 0.05, **P < 0.01, ***P < 0.001 (two‐way ANOVA).

The antitumor activity of phenformin has previously been attributed at least in part to its ability to activate AMPK. As such, we next assessed AMPK phosphorylation in liver cancer cells following phenformin treatment, revealing that AMPK phosphorylation was significantly enhanced in treated cells, consistent with our expectations (Fig. 3A,D). We then assessed the impact of Ku on this pathway, as ATM has been reported to either inhibit or activate AMPK in a context‐ or cell type‐specific manner [15, 16, 17]. In this study, we found that Ku treatment significantly enhanced AMPK phosphorylation (SMCC‐7721: 2.11 ± 0.36, Hep‐G2: 1.57 ± 0.09) without altering total AMPK levels (Fig. 3B,E). Combination phenformin and Ku treatment further enhanced p‐AMPK levels more effectively than did either single‐agent treatment, underscoring the synergistic activity of these compounds as regulators of AMPK pathway activation (Fig. 3C,F). We also assessed the activation of downstream p‐mTOR and p‐p70s6k levels in treated cells, revealing that both mTOR and p70s6k phosphorylation were effectively inhibited by phenformin, Ku, or synergistic combination treatment with both of these agents.

### Assessment of mitochondrial activity and energy status

**Fig. 4:**
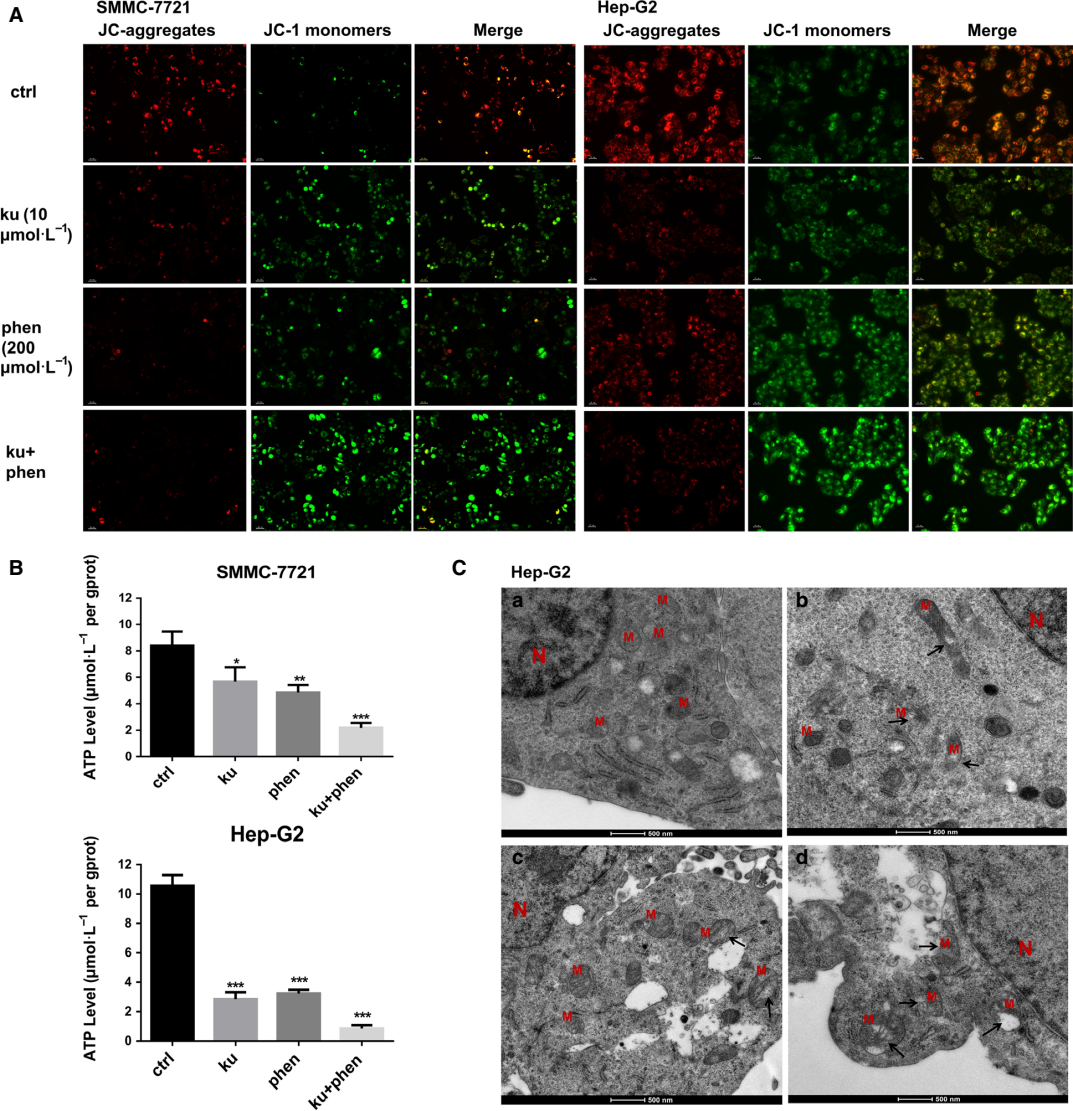
The impact of Ku and phenformin on mitochondrial metabolism in SMMC‐7721 and Hep‐G2 cells. (A) Ku and phenformin attenuated MMP in Hep‐G2 and SMMC‐7721 cells. Cells were exposed to Ku and phenformin for 24 h followed by a 20‐min incubation with JC‐1. When MMP (ΔΨm) was higher, JC‐1 accumulated in the mitochondrial matrix to form aggregates, producing red fluorescence. Otherwise, green JC‐1 monomer fluorescence was indicative of cells exhibiting a reduction in ΔΨm. Scale bar, 20 μm. (B) Ku (10 µm), phenformin (200 µm), or a combination of the two decreased ATP levels in SMMC‐7721 and Hep‐G2 cells. (C) 24 h after the treatment with Ku (10 µm) alone (B), phenformin (200 µm) alone (C), or a combination of the two (D), cellular ultrastructures were investigated using transmission electron microscopy in Hep‐G2 cells. Scale bar, 500 nm. Data are means ± SD, *n* = 3. *P < 0.05, **P < 0.01, ***P < 0.001 (one‐way ANOVA).

Prior studies have found that phenformin functions as an inhibitor of mitochondrial complex I, thereby impacting mitochondrial metabolism. In liver cancer cells, phenformin‐mediated growth inhibition has been linked to mitochondrial fragmentation [18]. As mitochondria are key producers of ATP, ATP levels can serve as a readout for mitochondrial functionality. We thus next assessed ATP production in both of these liver cancer cell lines following treatment, revealing that both phenformin and Ku impaired ATP production in these cells, compared with the blank control group, the ATP level in cells treated with both phenformin and Ku decreased by two to three fold (Fig. 4B). To evaluate the relationship between reduced ATP levels and cell death, we then assessed MMP using the JC‐1 fluorescent probe. We found that a 24 h treatment with phenformin or Ku was sufficient to increase the levels of monomeric JC‐1 and decrease JC‐1 aggregation within liver cancer cells (Fig. 4A), consistent with a reduction in MMP. Importantly, combination treatment with both phenformin and Ku further decreased MMP in treated cells, further underscoring the synergistic activity of these two compounds.

### Phenformin and Ku synergistically induce mitochondrial damage

Lastly, we assessed mitochondrial morphology in liver cancer cells at 24 h post‐treatment with phenformin and Ku, revealing a range of ultrastructural changes consistent with damage. Notably, mitochondria in cells treated with both of these agents appeared hollow with only an outer membrane remaining visible, whereas this was not observed in any cells treated with either agent alone (Fig. 4C).

## Discussion

As it is more readily absorbed by tumor cells, the anticancer activity of phenformin is nearly 50 times superior to that of metformin [19]. However, few studies to date have explored the antineoplastic activity of phenformin. Many reports have suggested that benzobiguanides can be effectively used to treat a range of different cancer types [20, 21]. Phenformin can inhibit mitochondrial respiratory chain complex I, thereby decreasing ATP synthesis and enhancing the AMP:ATP ratio within cells, ultimately inducing AMPK activation [22, 23]. AMPK serves as an intracellular energy sensor that controls metabolic activity in a dynamic manner [24]. Some research suggests that AMPK is a key target of biguanides and is activated in the liver in response to biguanide treatment [18].

Prior studies of fibroblasts in individuals affected by ataxia–telangiectasia syndrome have shown that defects in ATM are closely associated with mitochondrial dysfunction [25]. While most studies of the anticancer activity of ATM inhibitors focus on the requirement for this protein in the context of DNA repair, we herein found that the antiproliferative effect of Ku on liver cancer cells was associated with the role of ATM as a regulator of mitochondrial function. Specifically, we found that ATM inhibition using Ku reduced ATP levels, decreased MMP, and disrupted mitochondrial morphology in treated tumor cells. Ku has also been previously shown to increase AMPK phosphorylation through undefined mechanisms [17, 26], consistent with our results. Whereas prior studies found that Ku reduced metformin‐induced AMPK phosphorylation, we instead observed significantly enhanced AMPK activation in cells treated with phenformin and Ku, together with the inhibition of downstream mTOR and p70s6k phosphorylation. ATM localizes to the mitochondrial compartment and is related to the maintenance of mitochondrial homeostasis [26, 27]. Reduced ATM phosphorylation corresponds to decreased mitochondrial respiration and cytochrome c oxidase activity [28]. Reduced ATM phosphorylation may induce mitochondrial uncoupling that eventually results in the impaired synthesis of ATP and an increased oxygen cost per unit of ATP production [27]. In this study, we found that Ku was able to simultaneously inhibit ATM phosphorylation and enhance AMPK phosphorylation, with both of these activities serving to inhibit the growth of cancer cells. However, phenformin increased both ATM and AMPK phosphorylation. Phenformin can activate ATM and repair double‐stranded DNA damage, which is not conducive to inhibiting the growth of tumor cells. Phenformin‐induced increases in ATM phosphorylation may suggest a decrease in phenformin sensitivity, similar to the observations reported by Nadkarni *et al*. [29]. Thus, a combination of phenformin treatment and ATM inhibition can block the repair of double‐stranded DNA so as to increase the antitumor effects of phenformin. Ku may additionally function through mechanisms associated with mitochondrial dysfunction and the inhibition of mitochondrial respiratory chain complex II [17], resulting in an increased AMP:ATP ratio and decreased intracellular ATP synthesis, thereby inducing AMPK activation.

Herein, we determined that as a single‐agent therapy, Ku was not able to suppress liver cancer cell proliferation in a highly efficient manner, whereas phenformin was an effective inhibitor of these tumor cells. Importantly, we determined that phenformin and Ku synergistically suppressed the proliferation of liver cancer cells, and we observed similar synergy with respect to the impact of these compounds on migration, ATP production, apoptosis, and mitochondrial morphology in these tumor cells. Mitochondria are key pharmacological targets in the context of cancer as they are essential regulators of proliferation and death [30]. Efforts to target mitochondrial metabolism have been employed as an approach to treating a range of cancers, including those dependent upon oxidative metabolism [31, 32]. When applied in combination, phenformin and Ku act upon mitochondria to impair ATP production, decrease MMP, alter mitochondrial morphology, and thereby activate AMPK. Overall, our findings indicate that a combination of phenformin and Ku can synergistically inhibit the growth and survival of liver cancer cells via suppressing mitochondrial metabolism and activating AMPK signaling.

## Conclusion

In conclusion, we found that Ku is an effective anticancer agent that can suppress the proliferation of Hep‐G2 and SMMC‐7721 cells through mechanisms associated with its ability to induce mitochondrial dysfunction and to enhance AMPK phosphorylation. Phenformin synergizes efficiently with Ku, highlighting this as a promising approach to treating liver cancer. Overall, these data provide a foundation for future *in vivo* analyses of the efficacy of these compounds against liver cancer, suggesting that this combination treatment strategy may have the potential to improve outcomes in patients affected by this deadly disease.

## Conflict of interest

The authors declare no conflict of interest.

## Author contributions

TW performed research, collected data, and wrote the original manuscript. MQ, JT, SZ, LH and XY conceived and designed the study, and had full access to all of the data obtained during the study.MH, JD, DX, XH, and JW take responsibility for the integrity of the data and the accuracy of the data analysis. GL and XY designed research and revised the manuscript.

## Supporting information


**Fig. S1.** The impact of phenformin on SMMC‐7721 and Hep‐G2 cell proliferation. Phenformin inhibited SMMC‐7721 and Hep‐G2 proliferation. SMMC‐7721 and Hep‐G2 cell viability was assessed at 72 h post‐treatment with phenformin alone. The error bars represent SD.

**Fig. S2.** The impact of phenformin and Ku on apoptosis in SMMC‐7721 and Hep‐G2 cells. (a) Representative flow cytometry scatter plots showing propidium iodide (*y*‐axis) and Annexin V‐FITC (*x*‐axis)‐stained cells. SMMC‐7721 cells were treated with 400 μm phenformin alone, 10 μm Ku alone, or both for 12 h. Hep‐G2 cells were treated with 200 μm phenformin alone, 10 μm Ku alone, or both for 24 h. (b) Quantification of flow cytometry experiments. Data are means ± SD, *n* = 3. *P < 0.05, **P < 0.01, ***P < 0.001 (one‐way ANOVA).

## Data Availability

The datasets used and/or analyzed during the current study are available from the corresponding author on reasonable request.
