# Supplementary figures and images for: Phenformin and ataxia‐telangiectasia mutated inhibitors synergistically co‐suppress liver cancer cell growth by damaging mitochondria
Source: FEBS Open Bio. 2021 Apr 3;11(5):1440–51. doi: 10.1002/2211-5463.13152 (PMC8091576; doi:10.1002/2211-5463.13152)

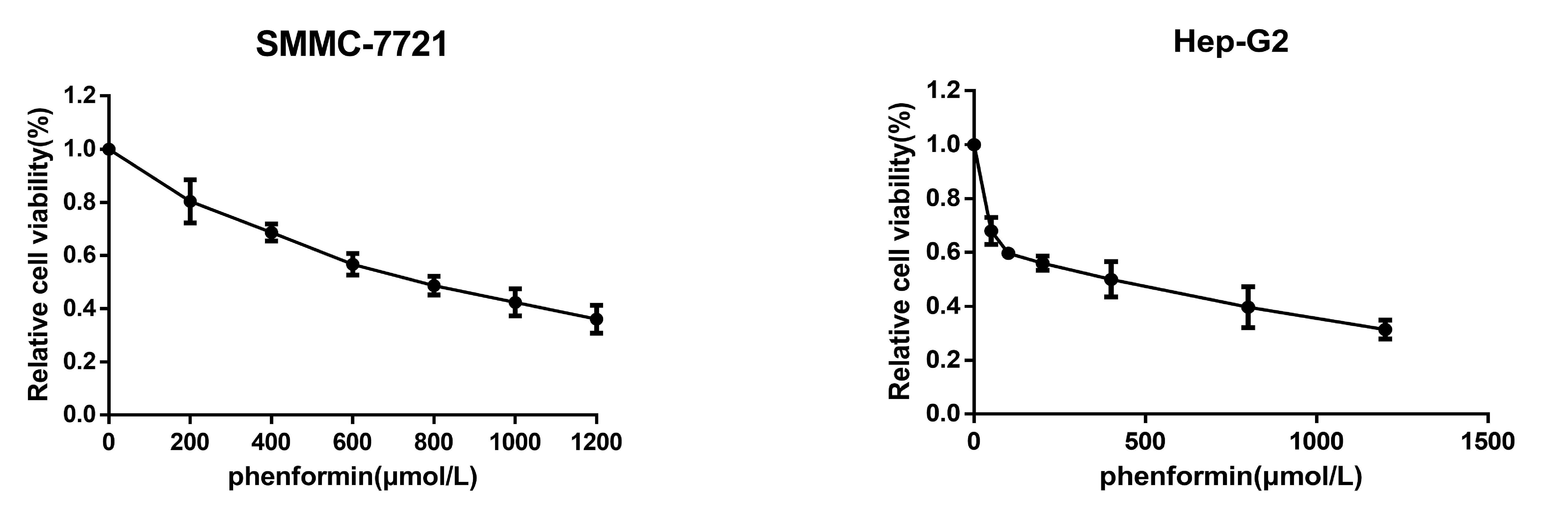

Supplement: Supplementary file 1 — Fig. S1. The impact of phenformin on SMMC‐7721 and Hep‐G2 cell proliferation. Phenformin inhibited SMMC‐7721 and Hep‐G2 proliferation. SMMC‐7721 and Hep‐G2 cell viability was assessed at 72 h post‐treatment with phenformin alone. The error bars represent SD. [file FEB4-11-1440-s002.tif]

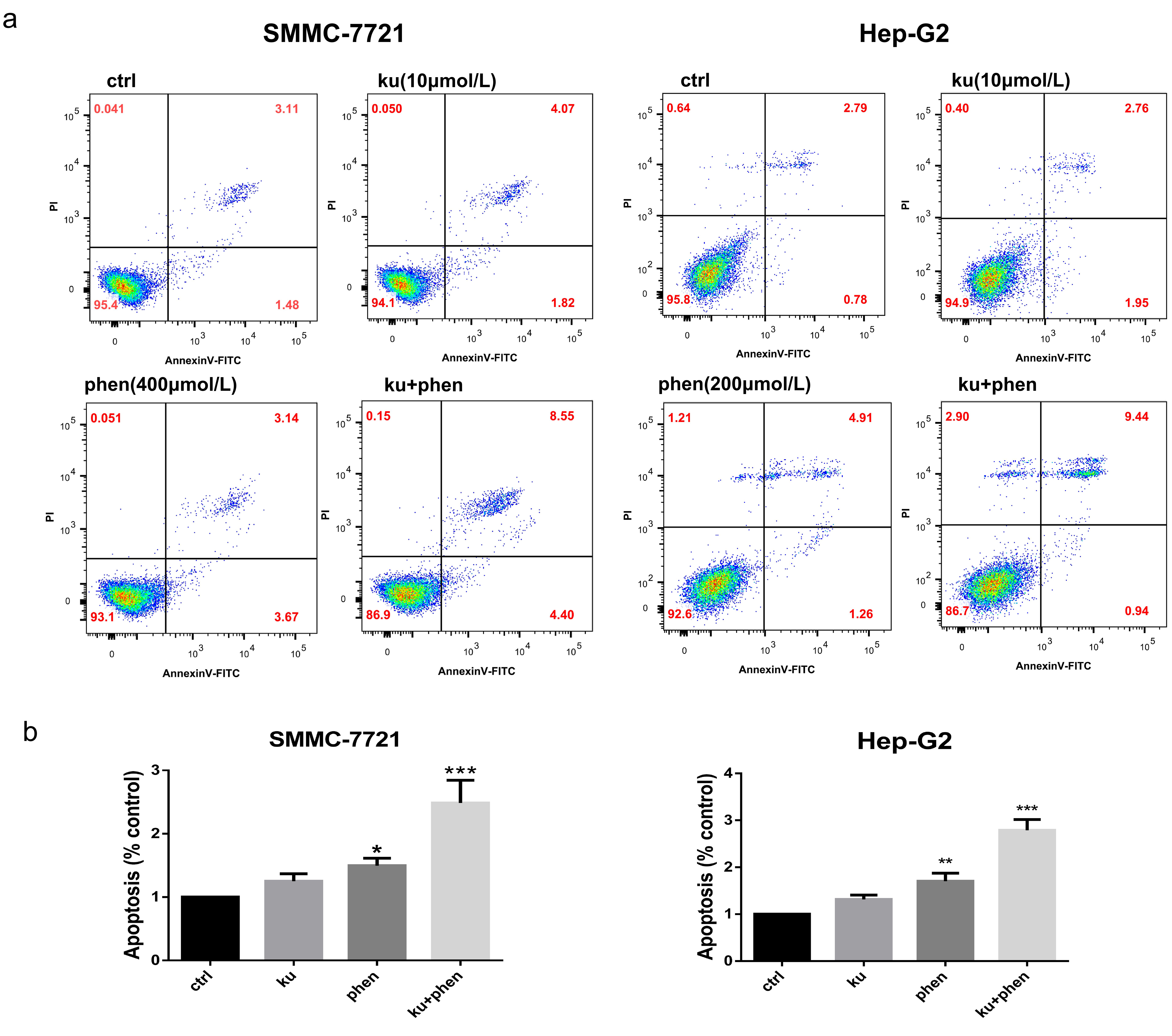

Supplement: Supplementary file 2 — Fig. S2. The impact of phenformin and Ku on apoptosis in SMMC‐7721 and Hep‐G2 cells. (a) Representative flow cytometry scatter plots showing propidium iodide (y‐axis) and Annexin V‐FITC (x‐axis)‐stained cells. SMMC‐7721 cells were treated with 400 μm phenformin alone, 10 μm Ku alone, or both for 12 h. Hep‐G2 cells were treated with 200 μm phenformin alone, 10 μm Ku alone, or both for 24 h. (b) Quantification of flow cytometry experiments. Data are means ± SD, n = 3. *P < 0.05, **P < 0.01, ***P < 0.001 (one‐way ANOVA). [file FEB4-11-1440-s001.tif]
